# Prevalence of functional cognitive impairment and associated factors in Brazilian community-dwelling older adults

**DOI:** 10.1590/1980-57642016dn11-010006

**Published:** 2017

**Authors:** Andréa Silva Gondim, João Macedo Coelho Filho, Alexandre de Andrade Cavalcanti, Jarbas de Sá Roriz Filho, Charlys Barbosa Nogueira, Arnaldo Aires Peixoto Junior, José Wellington de Oliveira Lima

**Affiliations:** 1 Departamento de Geriatria. Faculdade de Medicina da Universidade Federal do Ceara Fortaleza, CE – Brazil.

**Keywords:** aging, epidemiology, prevalence, elderly, dementia

## Abstract

The identification of the prevalence of cognitive impairment and associated factors among older adults is important in countries facing rapid demographic transition, given the significant implications for public policy and health planning.

**Objective:**

To determine the prevalence of functional cognitive impairment (FCI) and associated factors in Brazilian community-dwelling older adults.

**Methods:**

A cross-sectional study involving 461 elderly subjects residing in Fortaleza city, Ceará was conducted. Cognitive assessment was performed using three tests: the MMSE (Mini-Mental State Examination), VF (Verbal Fluency) and CT (Clock Test). The functional capacity evaluation was based on a survey of 21 basic and instrumental activities of daily living (ADLs). Cognitive impairment was defined by MMSE cut-off points adjusted for literacy. Functional impairment was defined as dependency to carry out more than four ADLs.

**Results:**

The prevalence of FCI was 13.64% (95% CI: 10.33 to 16.64%). FCI was proportionally associated with age with OR=2.24 (95% CI: 1.04 to 4.79) for individuals aged 70 to 79 years and OR=8.27 (95 % CI: 4.27 to 16.4) for those aged 80 to 100 years. FCI was associated with self-reported diseases including hypertension OR=2.06 (95% CI: 1.17 to 3.65), stroke OR=2.88 (95% CI: 1.66 to 5.00) and acute myocardial infarction OR=2.94 (95% CI: 1.59 to 5.42). The occurrence of FCI was proportionally correlated with the number of drugs used.

**Conclusion:**

Functional cognitive impairment is a prevalent condition in Brazilian community-dwelling older adults and its occurrence is associated with age, number of drugs used, and vascular morbidities.

## Abstract (pt)

A identificação da prevalência de déficit cognitivo, e fatores associados, é importante, dadas as implicações significativas para as políticas públicas e planejamento em saúde.

**Objetivo:**

Determinar a prevalência de CCF e fatores associados em idosos de uma área urbana brasileira.

**Métodos:**

Estudo transversal envolvendo 461 idosos residentes em uma área urbana de Fortaleza, Ceará. Foi aplicado um questionário socioeconômico e de saúde e realizou-se avaliação cognitiva através de três testes de rastreio: Miniexame do Estado Mental (MEEM), FV (Fluência Verbal) e TR (Teste do Relógio). A avaliação da capacidade funcional foi feita por um questionário de 21 atividades da vida diária. Comprometimento cognitivo foi definido pelo comprometimento no MEEM ajustado para escolaridade. Comprometimento funcional foi definido pela incapacidade para mais de 4 AVDs.

**Resultados:**

A prevalência de CCF foi de 13,64% (IC 95%: 10,33-16,64%). CCF foi proporcionalmente associado à idade: razão de chances (RC) 2,24 (IC95%: 1,04-4,79) para 70 a 79 anos e RC 8,27 (IC 95%: 4,27-16,4) para 80 a 100 anos. CCF apresentou associação significante com doenças autorreferidas: hipertensão arterial sistêmica apresentou RC 2,06 (IC 95%: 1,17-3,65), acidente vascular cerebral de 2,88 (IC 95%: 1,66-5,00) e infarto agudo do miocárdio de 2,94(IC 95%: 1,59-5,42). A ocorrência de CCF correlacionou-se com maior número de medicamentos usados.

**Conclusão:**

O CCF é condição comum entre os idosos no Brasil e sua ocorrência está associada à idade, ao número de fármacos utilizados e às morbidades vasculares.

## INTRODUCTION

Prevalence and incidence projections indicate that the number of people with dementia will continue to rise, particularly among the oldest old, and countries undergoing a demographic transition will experience the highest growth. Life expectancy in Brazil has been increasing rapidly since the 1940s and Alzheimer's disease (AD) has become a major public health concern as the world's population ages. The mean prevalence of AD in Brazil is higher than that found in the rest of the world, especially among women and the illiterate.^1^

The hallmark clinical phenotype of AD is a gradual and progressive decline in two or more cognitive domains, most commonly involving episodic memory and executive functions, which is sufficient to cause social or occupational impairment.^2^ Dementia causes a high burden of suffering for patients, their families and society. For patients, it leads to increased dependency and other comorbid conditions. Family caregivers show high rates of burden and psychological morbidity, as well as social isolation, physical ill-health, and financial hardship.^3-9^ Screening tests might be able to identify persons with undiagnosed dementia and thereby allow patients and their families to receive care at an earlier stage in the disease process.^10^ Cognitive function tests are very important in the diagnostic process and screening tools can be used in the primary care context both to give an indication of the extent of the cognitive problem and to monitor the level of cognitive performance over time.^11^

The Mini-Mental State Examination (MMSE) has been considered an important test to screen for cognitive impairment and dementia in the various clinical and community settings since 1975.^12^ One very important issue regarding the screening of dementia is the influence of educational level and cultural differences on some universally applied tests, such as the MMSE. Many studies have provided different cut-off scores for cognitive impairment according to age and educational level, both in developed and developing countries.^13^ Verbal fluency is also a very useful test, which can be used to evaluate executive functions and language, whereas the category test can be used to evaluate semantic memory.^14^

Dementia is characterized by a permanent and progressive or transient cognitive decline, caused by multiple etiologies, sufficiently intense to interfere in the person's professional and social activities. AD affects a person's ability to think, communicate, and the capacity to perform basic activities of daily living (ADL). The term "dementia" has been eliminated from the DSM-5 and replaced with "neurocognitive disorder", which is defined as cognitive decline from a previous level of performance in one or more of the cognitive domains, based on the concerns of the individual, a knowledgeable informant, or the clinician, as well as a decline in neurocognitive performance.^16^ The Scientific Department of Cognitive Neurology and Aging of the Brazilian Academy of Neurology published recommendations for the diagnosis and treatment, as well as for cognitive and functional assessment, of neurocognitive disorders. The diagnosis includes impairment in two of the following five cognitive domains: memory, executive function, language, visual-spatial ability or personality change.^17^ The diagnosis of dementia requires not only the presence of both cognitive and social or occupational impairment, but also decline from a previous level of functioning and the exclusion of other causes, including psychosis, depression and *delirium*. The diagnosis of dementia should therefore include clinical interview. Cases of impairment in cognitive and functional capacity identified by trained interviewers at home, without further clinical assessment, should be defined as Functional Cognitive Impairment (FCI),^15^ although it is a good correlate of dementia.

The aim of this study was to determine the prevalence of FCI and associated factors in Brazilian community-dwelling older adults.

## METHODS

**Subjects.** The current investigation was part of a survey conducted in the city of Fortaleza, Brazil, to assess the functional status of community-dwelling older adults. The population comprised elderly subjects (60 years or older) living in Rodolfo Teófilo neighborhood, the area surrounding the School of Medicine of the Federal University of Ceará. The elderly population of Rodolfo Teófilo area consisted of 1061 subjects and represented approximately 9% of the general population of the area. The participants were all interviewed at home by specially trained interviewers at the Center for Elderly Care, Federal University of Ceará. The interviewers were selected based on their age, educational level, good eloquence, responsibility, commitment, time availability and familiarity with the neighborhood. The socioeconomic characteristics and The Human Development Index of this neighborhood, classified as medium, are similar to most of the city's neighborhoods.^18^ A sample of 766 older subjects were randomly selected to be submitted to cognitive assessment in addition to functional assessment originally included in the survey. Of the initially selected subjects, 418 agreed to participate and were at home at the time of the interview. Owing to resource constraints, we were unable to carry out subsequent contact with older subjects who were absent on the first home visit. The study protocol was approved by the Ethics Committee of the Federal University of Ceará, under Protocols 155/08 and 591/08, and all participants signed the written free and informed consent form.

**Measurements.** A multi-dimensional questionnaire was applied to assess socioeconomic and health status. The following socioeconomic variables were analyzed: age, gender, educational level, marital status, income, presence of caregiver and whether the elderly lived alone or not (living arrangements). Health status was analyzed by the following variables: number of diseases, number of medications, number of hospitalizations, number of visits to the doctor, episodes of falls, weight loss and frequency of physical activity.

Cognitive assessment was carried out through three screening tests: the MMSE (Mini-Mental State Examination), VFT (Verbal Fluency Test) and CDT (Clock Drawing Test).^19^ Cognitive impairment was defined by the MMSE score with the following cut-off points: 28 points or more (individuals ≥8 years of education), 24 points (4-7 years' education), 23 points (1-3 years) and 19 points (illiterate individuals), plus abnormal CDT (scores of 1-10 points), or abnormal VFT (less than 8 or 9 names for those with <8 years of education) or less than 14 names for those with ≥8 years of education. Functional capacity (FC) included the various dimensions that affect the lives of the elderly and was assessed by a survey of 21 basic and instrumental activities of daily living.^21^ Functional impairment was defined as dependence to carry out more than four activities of daily living.^20-27^

**Statistical analysis.** Proportions were described by a points estimate and a 95% Confidence Interval. The Chi-square test (Fisher's exact test) and Prevalence Ratios were employed to evaluate associations between independent variables and cognitive and functional variables. Fisher's exact test was used when the expected values in any of the cells of a contingency table were below 5.

Associations were considered significant when the 95% Confidence Interval of the Odds Ratio did not contain the value 1.0. The analysis of the independent variables "Gender", "Age", "Status", " Activity Outside Home" and "Physical Activity" showed the association between each variable and Functional Cognitive Impairment (FCI). The independent variable "age" was analyzed as a continuous and categorical variable. Multivariate analysis was performed using logistic regression for independent variables. The objective was to estimate the association between Age and FCI, adjusted for potential confounders. Independent variables that were associated with FCI with a p-value ≥0.250 were considered "potential confounders".^28^

## RESULTS

The study revealed that 117 participants were male (27.99%) and 301 female (72.01%) and the majority (47.84%) were aged between 60 and 69 years. The proportion of females in the sample of subjects was higher than that found in the Rodolfo Teofilo area (55.6%), The same population was divided into two categories: 194 lived with a spouse (46.41%) and 224 did not live with a spouse (53.59%), which included single, divorced and widowed (Table 1) individuals.

**Table 1:** Sociodemographic characteristics of the study participants.

Characteristics		Total=418 n (%)
Gender	Male	117 (27.99)
	Female	301 (72.01)
Age (years)	60-69	200 (47.84)
	70-79	143 (34.21)
	80-100	75 (17.94)
Marital status	Married	194 (46.41)
	Divorced / widowed / single	224 (53.59)
Level of education (in years)	≤ 3 years	151 (36.12)
	4-7 years	120 (28.70)
	≥ 8 years	147 (35.17)
Household	Non-family	27 (6.46)
	Single-generation	71 (16.98)
	Two-generation	142 (33.97)
	Three-generation	152 (36.36)
	Others	26 (6.22)
Activity outside home	Yes	50 (11.68)
	No	368 (88.32)
Individual income (Reais)	Income ≤ R$ 415	240 (56.07)
	R$ 416 -R$ 1,245	100 (23.92)
	Income ≥ R$ 1,246	78 (20.00)
Family income (Reais)	Income ≤ R$ 800	215 (51.43)
	R$ 801 - R$ 1,400	84 (20.09)
	Income ≥ R$ 1,401	119 (28.44)
Physical activity	Yes	144 (34.45)
	No	274 (65.55

The study found predominantly (56.07%) low individual income, and household income ≥800 reais/month (51.43%) (Table 1). The most frequent household arrangement consisted of three generations living together (36.36%), followed by two-generation (33.97%) and single-generation (16.98%). The prevalence of households with only one person was only 6.46% (Table 1). A higher proportion of patients did not perform physical activity (65.55%) than performed it (34.45%) (Table 1). The study also analyzed the presence of comorbidities through the question: "Has any doctor or health professional ever told that you have or have had any of these diseases?". The most frequent diseases were hypertension in 250 (59.80%), arthritis in 119 (28.47%) and diabetes in 85 (20.33%) individuals. Parkinson's disease in 4 (0.9%) individuals was the least prevalent comorbidity. A total of 240 participants (48.80%) reported none or only one illness.

Data not shown in table

FCI prevalence according to the definition proposed in this study-MMSE + (CDT or VFT) + FC-showed a rate of 13.64% (95% CI, 10.33 to 16.64%) considering the elderly aged 60 years or older. Assuming the FCI definition as MMSE + VFT + FC, gave a prevalence of 7.89% (95% CI: 5.29 to 10.49). When FCI was defined as MMSE + FC, an association rate of 14.83% was determined (95% CI, 11.41 to 18.25) (Table 2). Functional Cognitive Impairment was analyzed using the age cut-off of 65 years or older for comparison with international studies. A total of 336 participants were included in the analysis; 82 participants aged between 60 and 64 years were not included. The prevalence of FCI thus changed to 15.48% (95% CI: 11.59-19.36) (Table 2).

**Table 2:** Prevalence of Functional Cognitive Impairment and of its cognitive and functional domains according to different measurements.

**FCI and instruments**	**60 years +**	
	**Prevalence (%)**	**95% CI**
Functional Cognitive Impairment	13.64	10.33-16.94
Mini-Mental State Examination	53.11	48.31-57.91
Clock Drawing Test	62.68	58.02-67.33
Verbal Fluency Test	26.79	22.53-31.06
Clock Drawing or Verbal Fluency Test	70.1	65.68-74.50
Mini-Mental State Examination and Verbal Fluency Test and Functional Capacity	7.89	5.29-10.49
Mini-Mental State Examination and Functional Capacity	14.83	11.41-18.25
**FCI and instruments**	**65 years +**	
	**Prevalence (%)**	**95% CI**
Functional Cognitive Impairment	15.48	11.59-19.36
Mini-Mental State Examination	52.98	47.61-58.34
Clock Drawing Test	65.77	60.67-70.87
Verbal Fluency Test	29.17	24.28-34.05
Clock Test or Verbal Fluency	73.21	68.45-77.97
Mini-Mental State Examination and Verbal Fluency Test and Functional Capacity	9.23	6.11-12.33
Mini-Mental State Examination and Functional Capacity	16.67	12.66-20.67

FCI: Functional Cognitive Impairment.

Age was the variable most strongly associated with FCI: the older the age, the higher the prevalence. The odds ratio for individuals aged 70-79 years was 2.24 (95% CI: 1.04 to 4.79) and for those aged 80-100 years was 8.27 (95% CI: 4.27-16.4). Being 'separated/widowed/single' was another variable associated with FCI, with an odds ratio of 2.43 (95% CI: 1.39 to 4.23). There was no statistically significant association between FCI and the other variables. However, there was a higher prevalence of females. The study also showed that the lower the individual's educational level, the greater the tendency for impairment, and the greater the number of generations in the household, the greater the tendency for FCI. Moreover, individuals with low income had more severe dementia.

Self-reported comorbidities were associated with FCI. 'Dementia' had an odds ratio of 5.71 (95% CI: 3.53 to 9.25), 'hypertension' of 2.06 (95% CI: 1.17 to 3.65), 'stroke' of 2.88 (95% CI: 1.66 to 5.00) and 'acute myocardial infarction' of 2.94 (95% CI: 1.59 to 5.42). There was no statistically significant association between other reported comorbidities. Additionally, a higher number of self-reported comorbidities was associated with a stronger tendency for FCI, with an odds ratio of 1.95 (95% CI: 1.15 to 3.33) for 2 or 3 comorbidities and an odds ratio of 5.14 (95% CI 2.73-9.69) for 4 or 5 comorbidities (Table 3).

**Table 3:** Prevalence of Functional Cognitive Impairment according to the presence of self reported comorbidities (n=418).

Characteristics		Total	Impairment		p-Value	Odds Ratio	
			N	%		Points	95% CI
Diabetes	Yes	85	15	17.7		1.39	0.82-2.39
	No	333	42	12.6	0.227	1	-
Parkinson's disease	Yes	3	0	0		-	-
	No	415	57	13.7	1	-	-
Dementia	Yes	10	7	70		5.71	3.53-9.25
	No	408	50	12.3	0	1	-
Hypertension	Yes	250	43	17.2		2.06	1.17-3.65
	No	168	14	8.3	0.01	1	-
Stroke	Yes	32	11	34.4		2.88	1.66-5.00
	No	386	46	11.9	0.002	1	-
Cancer	Yes	14	4	28.6		2.18	0.92-5.17
	No	404	53	13.1	0.109	1	-
Rheumatic diseases	Yes	119	18	15.1		1.16	0.69-1.94
	No	299	39	13	0.576	1	-
COPD	Yes	16	1	6.3		1	-
	No	402	56	13.9	0.708	2.23	0.33-15.10
Acute myocardial infarction	Yes	22	8	36.4		2.94	1.59-5.42
	No	396	49	12.4	0.005	1	-
Depression	Yes	50	8	16		1.2	0.60-2.39
	No	368	49	13.3	0.604	1	-
Number of diseases	None or 1	240	21	8.8		1	-
	2 or 3	158	27	17.1		1.95	1.15-3.33
	4 or 5	20	9	45	0	5.14	2.73-9.69

COPD: chronic obstructive pulmonar disease.

## DISCUSSION

This study of 418 elderly residents in the neighborhood of Rodolfo Teófilo, which assessed the prevalence of FCI and the factors associated with this condition was carried out in a Brazilian region with low socioeconomic status. The prevalence of FCI estimated in the study was 13.64%, considering elderly individuals aged 60 years or older, and 15.48% for the age of 65 years or older. These prevalence rates, which are relatively high, prove very similar to those found in other population-based studies in Brazil.^23,25,29^ The differences found for other data may suggest slight regional differences, but there were similarities in the proportion of elderly individuals with FCI in the studied areas. This finding is supported by the observation that the methodology used in the three studies was very similar at the first stage.

Some studies assessing the prevalence of dementia in the community were carried out in two phases, with the first being used to determine FCI and the second to establish the diagnosis using biochemical tests and imaging studies aiming to improve assessment and define the differential diagnosis.^23,25,30^ In the present study, only the first phase was carried out, precluding any estimates of the prevalence or types of dementia found in the study volunteers.^23,25^

There are some difficulties when comparing the aforementioned studies with the present investigation, as previous studies used only the MMSE as a cognitive screening test or employed other cognitive-only assessment tools. The screening tests used in these cases identify only cognitive impairment and provide a very generic diagnosis of dementia without considering functional impairment. Thus, these instruments may provide only a superficial evaluation, rendering the diagnosis inaccurate. The SABE study, carried out in São Paulo, found a FCI prevalence of 3.4%, but used only the MMSE for screening and diagnosis.^31^ A study carried out in Fortaleza twenty years ago found a rate of 8.4% for the prevalence of dementia in individuals aged 65 years or older. The study, in addition to being carried out in a single step, used the Information, Memory and Concentration (IMC) test in the screening phase of the study, with positive cases being classified as "senile dementia".^32^

The prevalence of dementia in international studies varies, with rates of 6.5% found in the Netherlands,^33^ 9.0% in Belgium,^34^ 7.0% in the USA,^35^ and 6.5% in Japan.^36^ Considering that these prevalence rates are similar to figures for Brazilian studies, with the exception of data for Nigeria, it may be inferred that the prevalence of FCI and dementia is similar in these populations and that the clinical pictures of dementia are fairly consistently distributed in many areas worldwide. It is possible to estimate, based on the data, that there is a worldwide prevalence of dementia of approximately 6.5% among the elderly population, which is of great relevance in terms of public health.

Many national and international studies have used only cognitive assessment as a screening tool. The results of these studies are more inconsistent, hampering adequate comparison with the present study.^37^ It should also be considered that many of these studies established the diagnosis of "dementia" or "Alzheimer's disease" based on the use of a single cognitive test, such as the MMSE. This finding suggests inadequacy when applying only the MMSE, even after correcting for educational level, as there is a tendency to produce many false positive results or to excessively increase sensitivity at the expense of specificity.^38,39^

Age was the only independent variable that showed a direct association with FCI in this study. This result has previously been confirmed in the literature, through the observation that age has an important influence on prevalence rates, with a two-fold increase every 5.1 years, showing that age itself is a risk factor for the development of dementia.^40,41^ Considering that Brazil is going through a rapid epidemiological transition, an alarming increase in the prevalence of dementia is expected among the population.^42-44^ Another variable that showed a possible association with FCI was the status of being single, widowed or separated. After multivariate analysis, this association was not confirmed due to the influence of the age factor.

The other variables showed only trends, albeit without statistical significance. The variables "educational level" and "individual income" have shown a controversial association in relation to dementia in the literature, but this relationship was not confirmed in the present study^45^. There was an attempt to adapt the screening tool used in the study to minimize the bias of educational level^46^ and thus, the results obtained in this study did not correlate educational level with FCI. The question remains whether the low educational level is in itself a risk factor for FCI or only a bias, given the difficulty individuals with lower educational level have when performing the cognitive tests.

The variable "gender" has also shown a questionable association with FCI and some earlier studies have shown a possible tendency for a higher prevalence of dementia among women.^47,48^ This study did not find statistical significance for the association, although women predominated among the individuals interviewed in the study (72.01%). Another variable that showed a tendency in relation to FCI, although not statistically significant, was household living arrangement: the higher the number of generations living in the same household, the greater the prevalence of impairment. This possible association has not been confirmed and more studies are required to clarify it.

Regarding the self-reported comorbidities, reporting of dementia, hypertension, stroke and acute myocardial infarction showed an important and significant association with FCI. Another study in Northeastern Brazil corroborated these results, showing hypertension and stroke as factors associated with dementia.^40^ Stroke was also strongly associated with FCI in a study carried out in the city of Ribeirão Preto, Sao Pauo State, Brazil.^23^ The presence of more than one morbidity also showed a strong statistical association with FCI, which leads us to believe that these patients care more for their health than those without FCI. Furthermore, patients using a higher number of drugs were also those with FCI. These findings demonstrate the need for differentiated health care for patients with FCI.

Among the limitations of this study is the fact that a significant number of subjects did not agree to participate or were not home at the time of the interview, which may mean that these individuals are more active and therefore have activities outside the home, thus being less often interviewed. This may have represent a selection bias, as this population is generally younger and has less dementia, which may have led to an overestimation of the prevalence of FCI in this sample. It also explains the predominance of females in the sample of subjects, with a much greater proportion than found in the Brazilian elderly population as a whole.

The study results show that functional cognitive impairment affects a significant portion of the elderly population and the magnitude of this public health problem needs adequate attention. Those affected by FCI are individuals who tend to be a burden to the community, their families and health care services. Many of them will develop advanced dementia, remaining bedridden for long periods and requiring continuous nursing care. These elderly will also be more vulnerable, sicker and use more medications, as well as demand more from health care services, as a result of their more severely affected health status.

The population is aging rapidly, therefore the consequences of this problem for public health and society will take on epidemic proportions. It must be considered that there are few public health actions addressing the needs of this population regarding preventive measures to control preventable risk factors associated with FCI. Additionally, the small number of specialized health care services for monitoring and treating this population and guiding the family and caregivers, makes the problem increasingly more significant.
